# A physical activity intervention for children with type 1 diabetes- steps to active kids with diabetes (STAK-D): a feasibility study

**DOI:** 10.1186/s12887-018-1036-8

**Published:** 2018-02-07

**Authors:** Helen Quirk, Cris Glazebrook, Holly Blake

**Affiliations:** 1 Centre for Sport and Exercise Science, Sheffield Hallam University, Collegiate Crescent Campus, Sheffield, S10 2BP UK; 2 Division of Psychiatry and Applied Psychology, Institute of Mental Health, Jubilee Campus, University of Nottingham, Triumph Road, Nottingham, NG7 2TU UK; 3 School of Health Sciences, A Floor, South Block Link, Queen’s Medical Centre, University of Nottingham, Nottingham, NG7 2HA UK

**Keywords:** Children, Feasibility study, Intervention, Paediatric diabetes, Physical activity, Process evaluation, Self-efficacy, Type 1 diabetes

## Abstract

**Background:**

This study describes the development and feasibility evaluation of a physical activity intervention for children with type 1 diabetes called ‘Steps to Active Kids with Diabetes’ (STAK-D). It aims to explore the feasibility and acceptability of the intervention and study design.

**Methods:**

Thirteen children aged 9–11 years and their parents were recruited from one paediatric diabetes clinic. A process evaluation was conducted alongside a two-arm randomised feasibility trial, including assessment of rate of recruitment, adherence, retention, data completion and burden, implementation fidelity and adverse events. Qualitative interviews with children (*n* = 9), parents (*n* = 8), healthcare professionals (*n* = 3) and STAK-D volunteers (*n* = 8) explored intervention acceptability. Interviews were analysed thematically.

**Results:**

Rate of recruitment was 25%, with 77% retention at 3-month follow-up. Study burden was low, data completion was high and the intervention was delivered as per protocol. No serious adverse event was reported. Engagement with intervention materials was generally good, but attendance at group activity sessions was low due to logistical barriers. Interview analysis identified preferred methods of recruitment, motivations for recruitment, barriers and facilitators to adherence, the experience of data collection, experience of the STAK-D programme and its perceived benefits.

**Conclusions:**

STAK-D was feasible and acceptable to children, their parents and healthcare professionals, but group sessions may present logistical issues. Recruitment and retention may be improved with a clinic-wide approach to recruitment.

**Trial registration:**

This trial was registered on ClinicalTrials.gov: NCT02144337 (16/01/2014).

**Electronic supplementary material:**

The online version of this article (10.1186/s12887-018-1036-8) contains supplementary material, which is available to authorized users.

## Background

United Kingdom (UK) guidelines recommend that children engage in at least 60 min of moderate-to-vigorous physical activity (MVPA) per day and muscle and bone strengthening activities on at least three days of the week [1]. In children with type 1 diabetes mellitus (T1DM), this level of physical activity can benefit glycaemic control [2], insulin sensitivity [3], protect against cardiovascular disease [4], and improve body composition [5], quality of life [6] and lifelong health. Yet figures suggest children with T1DM do not meet physical activity guidelines [7–10]. Possible barriers to physical activity include exercise-induced hypoglycaemia [11] or parental concerns about hypoglycaemia [12]. Parents of children with T1DM have perceived a lack of education around physical activity [12] and healthcare professionals (HCPs) have identified training needs to facilitate their role as promoters of physical activity to children with T1DM [13, 14].

The promotion of physical activity in children with T1DM requires an understanding of the underlying influences on behaviour which should draw upon psychological theory of behaviour change. However, a systematic review showed that previous physical activity interventions for children with T1DM have lacked a theoretical underpinning [15].

An existing theory-driven intervention targets children who may face unique challenges to physical activity [16]. Steps To Active Kids (STAK) targets children who have a chronic condition, low self-efficacy for physical activity, low levels of physical activity, or are overweight. It includes educational materials, a physical activity diary and pedometer, group activity sessions and goal-setting strategies using Motivational Interviewing (MI) techniques [17]. A cluster-randomised controlled trial in school children aged 9–11 found that STAK improved self-efficacy for physical activity and increased self-reported physical activity at 12 months follow-up [16] (Glazebrook et al., *under review*).

In the current study, we utilised findings from our formative research [12, 13, 15, 18] to develop STAK to meet the needs of children with T1DM. We aim to establish the feasibility and acceptability of STAK-D for children with T1DM aged 9–11 years. In this manuscript we report the feasibility trial and findings from an embedded qualitative study. As this is a feasibility trial, the sample lacks statistical power and we do not test effectiveness hypotheses. Instead, we descriptively evaluate the trial’s feasibility, acceptability and safety.

Study objectives

1. Demonstrate the feasibility of research processes; recruitment, adherence, retention and data collection.
2. Demonstrate the feasibility of intervention processes; delivery of the STAK-D programme and adverse events.
3. Conduct qualitative interviews with key stakeholders to explore the acceptability of the STAK-D programme.
4. Provide information that will inform interventions to promote physical activity among children with T1DM.

## Methods

### Participants and recruitment

#### Child-parent dyads

Children and their parents were recruited from a single paediatric diabetes clinic in the UK. Eligibility criteria were as follows:

- Children aged 9–11 years
- Diagnosed with T1DM for at least three months
- Able to understand spoken and written English
- Have a consenting parent or carer

A letter was sent to parents of potentially eligible children from the clinical team, inviting them to express their interest by return of a slip in the mail, or alternatively, parents were introduced to the researcher at their routine clinic appointment.

#### Healthcare professionals and STAK-D volunteers

Four healthcare professionals (HCPs) from the clinic had been aware of the research and were contacted by the researcher at the end of the study with an invitation for an interview. Three provided informed consent. A clinical support worker who assisted with study recruitment provided informed consent and was interviewed. Delivery of the STAK-D group activity involved eight volunteers (two or three volunteers present per session). These volunteers were pre-registered healthcare students. All volunteers were contacted at the end of the study, invited for an interview and informed consent was received from seven volunteers.

### Randomisation and blinding

Child-parent dyads were randomised after baseline assessments using numbered opaque sealed envelopes and a random number generator. The first three participants were randomised 1:1 to each study group, after which the allocation ratio was 2:1 in attempt to increase rate of recruitment to the intervention group. As this was a small feasibility study, the researcher who collected data (first author) also delivered the intervention and therefore was not blind to treatment allocation. Similarly, blinding of outcome assessors was not possible given research and resource restrictions.

### Treatment group allocation

The study was a two-arm randomised feasibility trial comparing STAK-D to usual care over three months.

### Usual care

‘Usual care’ in this context is difficult to assess, but our previous research suggests that physical activity promotion in current clinical management of paediatric T1DM is limited [12, 13].

### Intervention

Steps to Active Kids with Diabetes (STAK-D) is a six-week intervention for children aged 9–11 years with T1DM and their parents and is designed for implementation as an adjunct to usual clinical care. Children and parents are reminded that diabetes management should follow the advice provided by the child’s diabetes team. The STAK-D programme provides general advice around regular blood glucose monitoring (e.g., before, during and after physical activities and regularly throughout the day). It provides information about hypoglycaemia and how to manage hypo- and hyperglycaemia that are consistent with the education given to patients in clinic. It also provides general advice around healthy eating which has been approved by specialist diabetes dieticians, but it does not give guidance on carbohydrate counting. It combines educational, behavioural and cognitive-behavioural strategies to promote children’s self-efficacy for physical activity and daily physical activity level (Table 1). The theoretical framework for STAK-D draws upon Social Cognitive Theory [19] and the importance of self-efficacy, social support and goal-setting. Table 1 demonstrates the theoretical underpinnings of each intervention component.

**Table 1:** STAK-D programme content and theoretical underpinning

Weeks	Intervention content	Theoretical underpinning
1–6	Activity diary for children: physical activity advice for children with T1DM, recommendations (five ‘pieces’ of activity a day), safety information, physical activity log and step-count diary.	Knowledge Persuasion (education) Self-regulation; goal-setting, self-monitoring Mastery experience
1–6	Pedometer: tool to promote goal-setting and self-monitoring.	Self-regulation (self-monitoring and goal setting) Mastery experience
1–6	STAK street dance DVD: developed for the original STAK programme [16] and teaches children a street dance routine in 28 × 10-min sessions.	Vicarious reinforcement (role models) Mastery experience Social support
1–6	Group activity sessions: circuit training-style group activity session in a leisure room situated in the hospital supervised by STAK-D volunteers. Children given option to bring friend/sibling.	Vicarious reinforcement Mastery experience Social support Verbal persuasion
1, 3, 6	Motivational Interview (MI) and goal-setting: 1:1 session with the researcher at the child’s home to explore children’s perceptions and understanding of physical activity, readiness to change and goal-setting.	Readiness to change Social support Self-regulation (self-monitoring and goal setting)
1–6	Parents' Booklet: physical activity advice for safe participation aiming to educate and encourage parental involvement.	Social support Observational learning (role models)

### Outcomes to assess feasibility and acceptability

Outcomes to assess feasibility and acceptability explored rate of recruitment, adherence, retention, implementation fidelity, adverse events and data completion.

### Recruitment

Recruitment referred to those who consented to participate out of those eligible. A recruitment rate of between 25 and 40% would be considered reasonable based on similar research in this population [20, 21].

### Adherence

Adherence referred to the number of children using each component of the intervention, including attendance at group activity sessions.

### Retention

Retention was defined as the number of participants reaching the end of the STAK-D programme and completing all scheduled data collection compared to the number who started. A retention rate of at least 70% at each time point would be considered feasible based on similar studies in this population [20, 22].

### Implementation fidelity

Implementation fidelity referred to the evaluation of whether the intervention was delivered as per protocol.

### Adverse events

Adverse events experienced as a result of participation in the research were evaluated. A serious adverse event was defined as any serious negative outcome resulting from STAK-D participation.

### Data completion

Data completion was defined as the frequency counts of missing items at data collection periods. The criterion for feasibility was met if less than 10% of items on each questionnaire were missing; the likely threshold for imputation in a definitive trial [23]. Reasons for missing data were explored. To assess questionnaire burden, parents were asked to rate; i) the time taken for completion, ii) readability, iii) comprehensiveness, and iv) whether children required assistance.

### Outcome data collection

Outcome data were collected at time of consent (baseline; T1), six weeks after baseline (T2) and three months (T3) after baseline.

### Self-reported physical activity

Children’s self-reported physical activity level was measured with a physical activity questionnaire (PAQ). This was a revised version of an original [24] modified for use in the UK with children who have long-term conditions by Glazebrook and colleagues (2006) [25]. Children were asked to rate a range of activities on a three-point scale representing how much of that activity they did (none, a little, a lot) at three time points in the previous 24 h; today before school (22 items), yesterday after school (22 items), and yesterday during school (11 items). Scores were summed to form a total score for physical activities (possible range 41–123) and a total score for sedentary activities (possible range 14–42), with higher scores indicating greater physical activities and sedentary activities, respectively. The authors of the original questionnaire demonstrated good agreement between questionnaire responses and observed activities [24].

### Objective physical activity

Children’s objective physical activity was measured by accelerometer (Actigraph GT3X+, Pensacola, FL, USA) worn on the non-dominant wrist at baseline (T1) and T2. Feasibility and acceptability of the accelerometers were evaluated by exploring response rates, compliance rates, wear times and children and parents’ perceptions. Accelerometers were initialised using ActiLife 6 to collect data for seven consecutive days. A recording epoch of five seconds was used. Non-wear time, excluding sleep hours, was classified as periods of ≥60 min of zero values, with an allowance of up to two minutes of interruptions between 0 and 100 counts [26]. A valid day was defined as at least nine hours during the “wake hours” of 07.00–23.00. A minimum of three valid days was required for analysis. Accelerometer data were visually checked for compliance and non-wear time was removed before analysis.

### Self-efficacy for physical activity

The Children’s Self-Perceptions of Adequacy in and Predilection for Physical Activity (CSAPPA) scale [27] was used to measure generalised self-efficacy and attitudes towards participation in physical activity. The scale was designed by Hay (1992) for 9–16 year-olds to identify low self-efficacy for physical activity [27] and is described in detail elsewhere [16]. The CSAPPA scale has demonstrated high test-retest reliability and strong predictive and construct validity [27, 28].

### Data analysis

Descriptive statistics describe sample characteristics, recruitment rates, retention rates, rates of completion, attendance and adherence rates (frequencies, percentages, means and standard deviations). Outcome data were analysed using IBM Statistical Package for the Social Sciences (SPSS) version 22 (SPSS Inc., Chicago, IL, USA) and should be interpreted as feasibility data only. Objective physical activity was calculated as time spent in physical activity intensity categories according to cut-point thresholds provided by Chandler et al. [29]. MVPA was assessed by summing the time spent in moderate and vigorous physical activity. Change over time in MVPA was calculated as the difference between means at T1 and T2. To describe the association between MVPA and self-reported physical activity, Pearson correlation analyses were conducted. Due to the exploratory nature of the study, no hypotheses were made and a two-tailed analysis was conducted. Change in mean CSAPPA scores over time from T1 to T2 and T1 to T3 was calculated. The data were not powered to detect statistically significant differences between groups; instead the focus was on estimates of change scores and 95% confidence intervals for the difference between means. Participants who withdrew from the research were removed from post-intervention analysis, but retained for baseline assessment unless they requested withdrawal.

### Embedded qualitative study

The embedded qualitative study involved interviews with children, parents, HCPs and STAK-D volunteers at T3 to explore acceptability of the trial processes and intervention. Semi-structured interviews explored the acceptability of research processes and intervention delivery (see Additional files 1, 2, 3 and 4 for interview guides). All trial participants provided informed consent to be asked to take part in an interview with the researcher. Eight children (intervention *n* = 4, control *n* = 4), eight parents, three HCPs and eight STAK-D volunteers were interviewed either face-to-face or via telephone. One child responded to interview questions via a paper survey.

Thematic analysis was used to analyse the qualitative interview data [30]. NVivo version 10 [31] facilitated the organisation of qualitative data and the identification of quotations to illustrate themes. Participant groups (children, parents, HCPs and volunteers) were interviewed and analysed separately, but findings are presented together and verbatim quotes are used as supporting evidence with details of the respondent in parentheses (INT = intervention group, CONT = control group, VOL = volunteer, HCP = healthcare professional).

## Results

### Recruitment

**Fig. 1:**
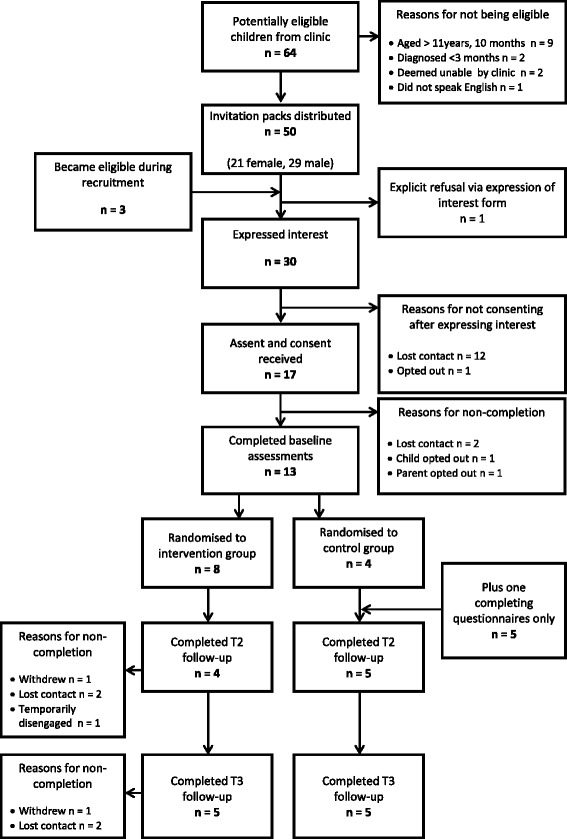
Flowchart of participants through the feasibility trial

Fifty-three child-parent dyads were identified from the clinic register as potentially eligible and were sent information about the research between May and August 2014. Of these, 30 expressed a desire for more information about the study (57% of those eligible). Reasons for refusal cited anecdotally included; i) already physically active, ii) other commitments, iii) current or recent involvement in other research, and iv) other medical conditions. Seventeen child-parent dyads (32% of those eligible) gave consent to participate. Contact was lost with two consenting participants and two withdrew prior to randomisation, giving a usable sample of 13 child-parent dyads (25% of those eligible) (Fig. 1).

Children had a mean age of 10.1 years (SD = 0.9 years) and had been diagnosed with T1DM for a mean duration of 51 months (4.3 years) (SD = 35.30 months); range = 5 to 127 months). Gender distribution across the whole sample was approximately equal (54% female). Twelve dyads agreed to be randomised with eight randomly allocated to intervention and five allocated to control (four randomised) (Table 2). After randomisation, groups did not differ on participant characteristics or outcome variables at baseline, except that the control group was all male (*n* = 5) and had a shorter length of diabetes diagnosis compared to the intervention group (Table 2 and Table 3).

**Table 2:** Participant characteristics at baseline

Outcome	Intervention	Control
Total (n)	8	5
Gender (n (%))	Male = 2 (25)	Male = 5 (100)
	Female = 6 (75)	Female = 0 (0)
Age (Mean (SD))	10.13 (.84)	10.00 (1.00)
Length of diagnosis in months (Mean (SD))	61.13 (37.29)	34.80 (27.77)
HbA1c (mmol/mol) (Mean (SD))	57.13 (10.25)	55.40 (11.78)
BMI (kg/m^2^) (Mean (SD))	19.51 (3.79)	20.49 (3.36)

**Table 3:** Physical activity and self-efficacy scores and change in scores over time

Outcome	Group	T1		T2		T3		T1-T2	T1-T3
		Mean (SD)	n	Mean (SD)	n	Mean (SD)	n	Difference (95% CI)	Difference (95% CI)
Accelerometer MVPA (mins)	Whole	84.82 (26.94)	11	69.46 (24.16)	8	-	-	−15.36 (−40.68, 9.96)	-
	INT	83.59 (27.25)	7	66.15 (18.67)	4	-	-	−17.44 (−52.49, 17.61)	-
	CONT	86.98 (30.42)	4	72.78 (31.38)	4	-	-	−14.20 (−67.67, 39.27)	-
Self-reported physical activity	Whole	54.10 (8.47)	13	49.63 (5.01)	8	54.30 (7.86)	10	−4.47 (−11.42, 2.48)	0.20 (−6.99,7.39)
	INT	56.78 (9.10)	8	52.00 (5.42)	4	58.80 (7.46)	5	−4.78 (−15.93, 6.37)	2.02 (−8.70, 12.74)
	CONT	49.80 (5.72)	5	47.25 (3.77)	4	49.80 (5.72)	5	−2.55 (−10.45, 5.35)	0.00 (−8.34, 8.34)
Self-efficacy Total	Whole	60.82 (7.10)	11	58.88 (9.49)	8	58.80 (9.14)	10	−1.94 (− 9.95, 6.07)	−2.02 (−9.46, 5.42)
	INT	61.71 (5.71)	7	63.50 (4.65)	4	58.60 (9.81)	5	1.79 (−5.84, 9.42)	−3.11 (−13.05, 6.83)
	CONT	59.25 (9.88)	4	54.25 (11.47)	4	59.00 (9.57)	5	−5.00 (−23.52, 13.52)	−0.25 (−15.64, 15.14)
Self-efficacy Adequacy	Whole	21.55 (3.11)	11	21.63 (3.78)	8	21.40 (3.44)	10	0.08 (−3.83, 3.99)	−0.15 (−3.64, 3.34)
	INT	22.43 (1.81)	7	23.50 (2.38)	4	21.60 (3.97)	5	1.07 (−1.79, 3.93)	−0.83 (−4.58, 2.92)
	CONT	20.00 (4.55)	4	19.75 (4.27)	4	21.20 (3.27)	5	−0.25 (−7.88, 7.38)	1.20 (−4.94, 7.34)
Self-efficacy Predilection	Whole	28.82 (4.12)	11	27.25 (4.80)	8	27.50 (5.28)	10	−1.57 (−5.90, 2.76)	−1.32 (−4.31, 1.67)
	INT	28.71 (3.99)	7	29.25 (2.75)	4	27.80 (5.36)	5	0.54 (−4.60, 5.68)	−0.91 (−6.89, 5.07)
	CONT	29.00 (4.97)	4	25.25 (5.97)	4	27.20 (5.81)	5	−3.75 (−13.25, 5.75)	−1.80 (−10.37, 6.97)
Self-efficacy Enjoyment	Whole	10.45 (1.57)	11	10.00 (2.07)	8	9.90 (1.91)	10	−0.45 (−2.21, 1.31)	−0.55 (−2.14, 1.04)
	INT	10.57 (1.72)	7	10.75 (1.50)	4	9.20 (2.17)	5	0.18 (−2.16, 2.52)	−1.37 (−3.87, 1.13)
	CONT	10.25 (1.50)	4	9.25 (2.50)	4	10.60 (1.52)	5	−1.00 (−4.57, 2.57)	0.35 (−2.05, 2.75)

*CI* confidence interval, *CONT* control group, *INT* intervention group, *MVPA* moderate-to-vigorous physical activity, *SD* standard deviation

### Adherence

The pedometer and activity diary were accessed by more children (n = 5) than the street dance DVD (*n* = 3) and group activity sessions (*n* = 4). One child attended 4/5 sessions, one child attended three sessions and two children attended twice. Reasons for attendance (or non-attendance) were explored in the interviews (see qualitative findings).

### Retention

The retention rate at T3 was 10/13 (77%): 5/5 in the control group and 5/8 in the intervention group. One child-parent dyad withdrew from the research and two were lost to follow-up.

### Implementation fidelity

All children in the intervention group received the STAK-D programme as per protocol. Motivational interviews (MI) took place with six individual children at their homes in week 1 or 2 of the intervention. Successful implementation of MI was dependent on home-visits which limited the frequency of sessions to one in-depth session per child due to the significant investment of time. Six group activity sessions were planned and five were delivered due to cancellation of the final session because of insufficient numbers of attenders.

### Adverse events

The researcher documented two episodes of hypoglycaemia (HbA1c < 4 mmol/L) during STAK-D group activity sessions. No other adverse event as a consequence of the STAK-D programme was reported.

### Data completion

Participants generally preferred to take the questionnaires home for completion as the clinic setting was time-pressured. Questionnaires were completed with little perceived burden. Most (*n* = 6) child-parent dyads took 11–20 min to complete the measures, four took less than 10 min and three took 20–30 min. Five parents reported that their child needed assistance to complete the questionnaires.

The CSAPPA scale data were visually scanned and single items were identified as missing at random for two participants. The mean of the subscale for that person was used (mean imputation). Of the 12 children asked to wear the accelerometer 11 agreed. At T1, all 11 children had complete accelerometer data (at least nine hours a day) for seven consecutive days (100% compliance). At T2, 8/11 children wore the accelerometer (one withdrew and two could not be contacted) of whom six had accelerometer data for seven days and two had data for five days (100% compliance to the three-day protocol criterion).

### Outcome data collection

Table 3 shows the change in mean physical activity and self-efficacy from T1 to T2 and also change in self-efficacy scores from T1 to T3.

### Self-reported and objective physical activity

Between T1 and T2, accelerometers detected a 15.4 min decline in MVPA, across the whole group on average. The decline was 17.4 min in the intervention group and 14.2 min in the control group. The correlation coefficient is described in terms of Cohen’s [32] classifications of effect sizes; .1 small, .3 moderate, .5 large. Children who had higher levels of MVPA as measured by the accelerometer had higher self-rated scores for physical activity (*r* = .568, *p* = .068; *n* = 11), which represented a large effect size, although not statistically significant.

### Self-efficacy for physical activity

From T1 to T2, the CSAPPA scale total self-efficacy score demonstrated a two point increase in the intervention group and a five point decrease in the control group. However, the improvement in the intervention group was not maintained to T3. The adequacy subscale followed a similar pattern, with the intervention group demonstrating an improvement from T1 to T2 that was not maintained at T3. Predilection scores remained relatively stable across all time points in the intervention group, whereas the scale detected a reduction in the control group’s predilection score over time (reduction of − 3.75 between T1 and T2). The enjoyment subscale remained relatively stable over time, except for a detected decrease between T1 and T3 in the intervention group (− 1.37).

### Qualitative findings

Qualitative analyses identified themes that closely matched the focus of the interview; which asked questions about trial procedures (recruitment and randomisation, adherence, data collection, and the intervention). Findings are supported by illustrative quotes in Additional file 5.

### Recruitment and randomisation

Four parents valued the invitation letter because they felt informed when later approached by a researcher in the clinic. Four parents preferred being approached by a researcher in clinic. Children in the intervention and control groups were motivated to participate in the research by their interest in physical activity and being healthy. Parents in both groups valued the chance to gain feedback into how active their child was and its effects on blood glucose levels. Two parents were personally motivated to participate in the research for its potential to encourage their child to be more physically active. Four parents described being motivated by the opportunity to help towards advancing knowledge about T1DM.

The HCPs expected higher recruitment, but acknowledged that, *“it’s quite a difficult client group to target”* (HCP02, Nurse)*.* The clinical support worker believed recruitment was low because children with T1DM are “*bombarded*” with research opportunities. All three HCPs suggested they could have promoted the research more. Consistent with this, all parents said the diabetes team had not discussed the research with them. Twelve out of thirteen participants were willing to be randomised and all those randomised reported satisfaction with the group they were allocated to.

### Adherence

Reasons for lack of adherence to the STAK-D programme were explored. One child was deterred because the programme only targeted children with T1DM, which echoed concerns about stigma raised by participants in our preliminary research. The HCPs believed that children’s adherence to the intervention was dependent on parental engagement and “*commitment from the whole family*” (HCP02, nurse). Likewise, parents perceived their busy lifestyle to be the main barrier to attendance at the group physical activity session e.g., *“our life is so busy… if we could’ve made it, we would’ve loved to have come”* (P01, mother, INT)*.* One mother implied that living with diabetes made it difficult to afford the time to do extra activities at the weekend (see Additional file 5). Two parents perceived the distance required to travel to the group activity session to be a barrier. The average (mean) distance the participants travelled to clinic was 10.3 miles (range 3.3 to 24.3 miles).

All five children who completed the intervention reported using the pedometer, although adherence to the pedometer was not measured explicitly. Three children engaged with the street dance DVD and found it enjoyable, two children did not use the DVD at all. The main reason for not engaging with the DVD was the child’s existing dislike of dance.

Facilitators to intervention adherence were: i) enjoyment, ii) bringing a friend or sibling, and iii) family engagement. Children’s enjoyment of physical activity motivated them to adhere. Every parent and STAK-D volunteer perceived the intervention to be fun and considered enjoyment to motivate children’s adherence. Three parents described family engagement with the STAK-D programme. One mother described how family members had worn a pedometer to compare activity levels, another described how they had substituted the street dance DVD for active video games as a family and a father described sibling involvement with home-based physical activities. Among the STAK-D group session attenders, all except one child chose to attend with a friend or sibling. This was generally perceived to facilitate attendance, but one volunteer suggested it created a division when participants attended the session alone.

### Retention

The primary motivator for continued participation among children in the control group was to use the accelerometer results *“to see how active”* (P07, male, INT) they were. Parents in both groups felt motivated by the objective feedback they would receive about their child’s level of physical activity. Additionally, two parents in the intervention group attributed their continued engagement to the low burden of the research processes (e.g., the researcher making home-visits).

### Data completion

Eight children gave positive feedback about wearing the accelerometer. When asked what they did not like about the accelerometer, three spoke about the wrist-strap being uncomfortable, one boy did not like other children asking what the device was, whereas another child “*liked telling people [about it]*” (P01, female, INT). Eight parents gave positive feedback about the accelerometer, describing it as “*brilliant*” (P02, mother, INT), “*good*” (P09, father, INT) and “*really interesting*” (P04, mother, CONT).

### The intervention

Parents described benefits the STAK-D programme. All parents perceived the information about physical activity to be beneficial for learning about the importance of physical activity and how it relates to blood glucose levels. Some felt that the information would be better suited to less informed families. All the parents perceived the physical activity data from accelerometers could help with diabetes management.

The pedometer was an optional part of the STAK-D programme and parents valued it for facilitating goal-setting, such as step-count targets. One mother believed that pedometers could help support clinical education about the relationship between physical activity and blood glucose control.

There were practical constraints to delivering the group physical activity sessions, but the activities involved were evaluated positively by those who attended. Children benefited through enjoyment of the novel activities. Parents valued the peace of mind of having STAK-D volunteers trained in diabetes management. One father valued his daughter and her sibling learning new activities and practicing them at home. A boy valued having fun with his friend*.* And his mother valued the insight it gave her son’s friend into, *“what things are like for children with diabetes”* (P07, mother, INT). All volunteers gave a positive evaluation of the STAK-D group session and organisation.

All HCPs valued the feedback they had received from the researcher about the group activity sessions because it gave insight into how children sometimes failed to demonstrate adequate blood glucose management. It was apparent at the activity sessions that some children and parents lacked an understanding of the importance of testing blood glucose levels pre and post exercise and failed to bring snacks to treat hypoglycaemia. In response to this, HCPs believed that future implementation of the group sessions would benefit from *“ground rules and expectations”* from the diabetes team about blood glucose testing, including, “*A statement from the doctor to say…these are some recommendations… you will test beginning, during and end, something just to make it more formal*” (HCP03, Dietician).

Parents described becoming more aware of their child’s physical activity level and one parent suggested it encouraged discussion with school teachers about physical activity. The HCPs positively appraised STAK-D for combining home-based and group-based activities, because it encouraged social support networks among parents and parental engagement. In agreement, parents described how STAK-D had prompted family-oriented physical activity.

## Discussion

Points relating to the feasibility of research processes and those relating to acceptability of the intervention will be discussed, before outlining the practical implications of the findings.

### Feasibility of research processes

It is possible to recruit children with T1DM and their parents to a physical activity intervention, although recruitment remains challenging as found in similar research with this population [20, 33]. Direct, in-person recruitment strategies were most effective as shown in other studies with parenting interventions [34]. Recruitment would benefit from techniques to translate participants’ initial expression of interest into consent. Studies need to focus on strategies to ensure that clinics engage with the research and promote participant recruitment.

Many of the children reported an existing interest in physical activity and so the sample may have been biased towards those who were already active. However, just under half of the children in this sample had low self-efficacy for physical activity, based on their predilection score of ≤27, a threshold used in a previous study [21], implying that there was potential for improvement. It remains a challenge to recruit those children who may be most in need of a more active lifestyle. For parents, being motivated by the personal relevance of the intervention is consistent with a previous study of a physical activity parenting course [34]. Some parents were attracted to the study by the potential for gaining feedback on the relationship between their child’s physical activity and blood glucose fluctuations, which highlights the need for physical activity resources for families [12, 13].

The sample size was modest, although reasonable to address the feasibility aims and is comparable with other research targeting this population [20, 35, 36]. Almost all participants confirmed their willingness to be randomised and the treatment groups were broadly balanced according to baseline characteristics except for the control group being all male. Overall retention in the control group was good, with a zero attrition rate. In the intervention group, retention rate was considered to be acceptable based on similar research [20, 22]. All children and parents desired feedback about the child’s physical activity level, which suggests that this could be used in future research to encourage uptake and continued participation.

Parents and children did not find the assessment procedures burdensome. Home-visits were considered a successful method of data collection. The CSAPPA scale and accelerometer were considered feasible, acceptable and able to detect change in outcomes over time. Compliance to the accelerometer protocol was acceptable at two time points, but suggested compliance may decline with the number of measurement episodes across a study. The accelerometer measure of MVPA correlated strongly with the self-reported physical activity data, suggesting agreement between the objective and self-report measures. The results support the utility of accelerometers for measuring what children recognise and contextualise as being physical activity. It also suggests that 24-h recall questionnaires might be a feasible method of physical activity measurement in children aged 9–11 years, and could be used to supplement objective data to provide information about the types of activities children participate in (e.g., organised sports, free play, active transportation).

### Intervention acceptability

Motivational Interview (MI) techniques elicited children’s values, beliefs and outcome expectations around physical activity and gained insight into the children’s perceived barriers and facilitators to goal attainment. In future delivery, more time should be allocated to MI and regular sessions should be scheduled with children to monitor and reassess their goals. Whilst home-visits for MI were feasible in this small-scale study, time and resource constraints of home-visits would need to be considered in a large-scale trial. Parents perceived the pedometer to facilitate the child’s self-monitoring, goal-setting and diabetes management, suggesting that more emphasis could be placed on activity tracking in future studies.

The STAK-D activity diary was well received by children and their parents. Pedometers and step-count logs promoted self-monitoring of daily step-count and activity behaviours. Children showed less interest in the educational elements and some parents felt the information was pitched for a less-informed audience. This suggests that information-giving could be better tailored to enhance individual impact.

The street dance DVD was not used by children who had no existing interest in dance, suggesting that the dance DVD should be demonstrated to children prior to its implementation or that techniques to engage children in more diverse physical activities should be explored.

Attendance at the STAK-D group activity sessions was poor, although comparable with attendance rates in a previous study implementing a structured education programme for children with diabetes [37]. Perceived benefits of the group activity session included the opportunity for children to practice and develop competency in new skills and for children have fun and be active with friends, which supports previous findings [12]. Reasons for non-attendance were logistical (i.e., session timing and location) rather than being related to the appeal of the session. The group activity sessions were valuable for HCPs to gain an insight into children’s diabetes management. Future implementation would benefit from firmly established blood glucose testing ground rules to support the promotion of optimal diabetes management behaviours.

Overall, most participants perceived STAK-D to be beneficial. This evaluation indicates that it is feasible to deliver STAK-D primarily as a home-based intervention with complementary group physical activity sessions, but the intervention in its current form requires some alterations to optimise its efficiency and potential efficacy. The next section provides information that will inform further development and implementation of interventions.

### Recommendations for a future trial

A key strength of this study is that the findings can be used to inform the design, development and implementation of a larger trial to explore the efficacy of STAK-D to promote self-efficacy and physical activity in children with T1DM. Here we address the main implications for; i) recruitment, ii) retention and adherence, and iii) intervention implementation.

### Recruitment

In this feasibility study, face-to-face recruitment was more successful but places burden on researchers. Increased study promotion and endorsement by the wider clinical team may enhance recruitment rates. The need for greater ‘buy-in’ from the wider clinic team to facilitate recruitment has arisen from similar research implementing a group-based programme for children with T1DM [37]. Future research could adopt a team approach, with the clinic staff working towards recruitment targets.

### Retention and adherence

Parents and children requested the results from the accelerometer immediately after the device was worn, but data could not be provided until the end of the study. Using accelerometer data as an incentive might encourage ongoing engagement and adherence, but may confound research findings. Post-programme maintenance strategies may be needed to maintain any beneficial effects and participants’ interest after cessation of the intervention. These may include “top-up” sessions [38] or the provision of continuing, tailored support such as a telephone helpline [39] and personalised letters [40]. During development of STAK-D there was no consensus from advisors on the best time to schedule the group activity sessions. A time when children are already attending clinic might enhance accessibility and eliminate additional hospital visits, but this would require extensive administrative planning. Planning sessions in school holidays may also increase uptake.

### Implementation

Implementing ground-rules for blood glucose testing during group activity sessions may promote management behaviours that meet clinic expectations. Providing family members with pedometers may encourage family involvement.

The accelerometer data could be used as an intervention tool in combination with blood test results to educate children and parents about blood glucose control in relation to physical activity. This may also promote health professionals’ engagement with activity monitoring if outcomes were shared with the clinic.

### Evaluation

This study gave insight into the feasibility and acceptability of STAK-D for children with T1DM. The mixed methodology gave insight into potential active ingredients as well as the diverse perspectives of participants. To the authors’ knowledge, this is the first research to demonstrate that wrist-worn accelerometers are acceptable among pre-adolescent children with T1DM.

Methodological limitations should be considered when interpreting the results. The researcher (first author) collected the data, delivered the intervention and conducted interviews, thus findings should be considered with potential for bias. An independent interviewer would strengthen the design of the study. Attention should be given to the potential for bias in the study sample. The small sample and limited uptake to the study may have resulted in a sample that was motivated and so over-estimating the acceptability of the intervention. Furthermore, participants allocated to the control group were all male despite randomisation. Usual care was not systematically assessed as part of this feasibility study, but should be monitored following recommendations by Erlen et al. (2015) [41].

## Conclusions

STAK-D was shown to be a promising intervention for children aged 9–11 years with T1DM. The intervention and research process were acceptable to children and their parents and evaluated favourably by HCPs. Changes are proposed to the research and intervention processes to optimise acceptability and efficacy of future implementation.

## Additional files

Additional file 1: Post intervention qualitative interview guide CHILDREN. (DOC 51 kb)

Additional file 2: Post intervention qualitative interview guide CLINIC HCPS. (DOC 34 kb)

Additional file 3: Post intervention qualitative interview guide PARENTS. (DOC 37 kb)

Additional file 4: Post intervention qualitative interview guide VOLUNTEERS. (DOC 33 kb)

Additional file 5: Quotes to support the themes in the embedded qualitative study. (DOCX 17 kb)

## Abbreviations

CONT: Control group
CSAPPA: Children’s Self-Perceptions of Adequacy in and Predilection for Physical Activity scale
HCP: Healthcare professional
INT: Intervention group
MI: Motivational interview
MVPA: Moderate to vigorous physical activity
STAK-D: Steps to active kids with diabetes
T1DM: Type 1 diabetes mellitus
VOL: Volunteer
