# Supplementary material for: A physical activity intervention for children with type 1 diabetes- steps to active kids with diabetes (STAK-D): a feasibility study
Source: BMC Pediatr. 2018 Feb 7;18:37. doi: 10.1186/s12887-018-1036-8 (PMC5804086; doi:10.1186/s12887-018-1036-8)

**Steps To Active Kids (STAK) Programme: Feasibility Study**

**Qualitative Script: Acceptability, Desirability and Feasibility**

**Children**

Participant ID……………………………………….

Date of Interview………………………………….

**CONTROL AND INTERVENTION GROUP**

1. At the beginning we sent out an information pack about the study to your parents, do you remember whether they talked to you about it and did they ask you whether you wanted to participate?
2. What did you think about the sound of the research/project?
3. Do you remember completing the questionnaires?
   1. Was there anything that was not so good about completing them?
4. Do you remember wearing the activity monitor?
   1. What did you think about it?
   2. Why did you like / not like wearing it?
   3. What do you think about having to wear it again?
   4. *If appropriate*: Probe **why** participants are interested in finding out the results?
5. Would you recommend this research to other children?
6. Do you have any questions?

**INTERVENTION GROUP ONLY**

1. How much did you use the STAK programme?

Did not use it Used it a bit Used it once a week Used it every day

1. What do you think about the STAK programme and the things included in it?
   1. STAK diary
      1. Did you read it / use it?
      2. What was it telling you?
   2. Dance DVDs
      1. Did you look at the DVD?
   3. Pedometer
      1. Did you wear it?
   4. Group sessions
      1. Did you attend?
      2. Probe why they did/did not attend (e.g. location, time)
      3. What did you like about the group sessions (or idea of a group if they could not attend)?
         1. Probe: other children – with diabetes/without diabetes
      4. What did you not like about the group session?
2. What did you think was particularly good about the STAK programme?
   1. Content
   2. Format
3. What would you change about the STAK programme to make it better?
   1. Content
   2. Format (e.g. paper diary)
4. Would you recommend this intervention to other children?

Yes No

Probe why? / how come?

Anything else you would like to say about this project or the STAK programme?

Any questions?


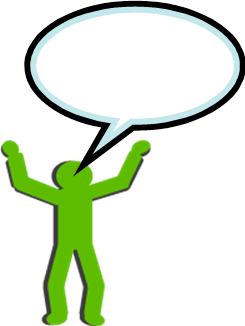

Supplement: Supplementary file 1 — Post intervention qualitative interview guide CHILDREN. (DOC 51 kb) [file 12887_2018_1036_MOESM1_ESM.doc]
