# Supplementary material for: A physical activity intervention for children with type 1 diabetes- steps to active kids with diabetes (STAK-D): a feasibility study
Source: BMC Pediatr. 2018 Feb 7;18:37. doi: 10.1186/s12887-018-1036-8 (PMC5804086; doi:10.1186/s12887-018-1036-8)
Supplement: Supplementary file 2 — Post intervention qualitative interview guide CLINIC HCPS. (DOC 34 kb) [file 12887_2018_1036_MOESM2_ESM.doc]

**Steps To Active Kids (STAK) Programme: Feasibility Study**

**Qualitative Script: Acceptability, Desirability and Feasibility**

**Healthcare professionals**

Participant ID……………………………………….

Date of Interview………………………………….

**** RECEIVE CONSENT BEFORE STARTING THE INTERVIEW ****

- - Have you read the information sheet?
  - Have you had the chance to ask any questions?
  - Are you happy to go ahead with the interview?

1. Overall, what do you think about this research?
2. What do you think about the STAK programme and the things included in it?
   1. STAK diary
   2. Dance DVDs
   3. Pedometer
   4. Parents resource
   5. Group sessions
3. Why do you think children/parents may not have engaged with the research?
   1. Explain the recruitment process.
   2. How we can minimise this in future work?
   3. Could we have done anything differently?
4. Do you think there were any negative effects of the STAK programme?
   1. Could we have done anything differently?
5. What did you think was good about the STAK programme?
   1. Content
   2. Format
   3. Effects on children/parents
   4. Its potential impact/effects
6. What would you change about the STAK programme to make it better?
   1. What additional information, if any, should be included?
   2. Content
   3. Format (e.g. paper diary, delivery/location)
7. What, if anything, have you/your clinic learnt from being involved in this research?
8. Can you suggest other ways that could be used to provide information about physical activity for children with Type 1 diabetes and their parents?
   1. What role do you think health professionals have in this kind of intervention?
9. Would you recommend this intervention to children?

Yes No

Why?

(if appropriate) What stage of diagnosis / age of child?

• Is there anything else we could we have done differently?

Anything else you would like to say about this project or the STAK programme?
