# Supplementary material for: A physical activity intervention for children with type 1 diabetes- steps to active kids with diabetes (STAK-D): a feasibility study
Source: BMC Pediatr. 2018 Feb 7;18:37. doi: 10.1186/s12887-018-1036-8 (PMC5804086; doi:10.1186/s12887-018-1036-8)
Supplement: Supplementary file 3 — Post intervention qualitative interview guide PARENTS. (DOC 37 kb) [file 12887_2018_1036_MOESM3_ESM.doc]

**Steps To Active Kids (STAK) Programme: Feasibility Study**

**Qualitative Script: Acceptability, Desirability and Feasibility**

**Parents**

Participant ID……………………………………….

Date of Interview………………………………….

**** RECEIVE (ongoing) CONSENT BEFORE STARTING THE INTERVIEW ****

- - Do you understand why we want your feedback?
  - Have you had the chance to ask any questions?
  - Are you happy to go ahead with the interview?

**CONTROL AND INTERVENTION GROUP**

1. What attracted you to taking part in this particular research study?
2. What did you think about the recruitment process?
   1. Receiving a letter at home
   2. Meeting Helen in clinic/speaking to Helen about it
3. Did you ever discuss the research with the diabetes team at the hospital?
   1. If appropriate: Probe what/when it got discussed
4. What did you think about us randomly putting your child into the group that got the STAK programme or the group that didn’t (random allocation)
   1. Were you happy with the group your child got put into?
5. What did you think about filling in the questionnaires?
6. What did ***you*** think about the accelerometer/activity monitor?
   1. What did ***your child*** think about the accelerometer?
   2. Why do you think they liked/did not like wearing it?
   3. *If appropriate*: Probe **why** participants are interested in finding out the results?
7. Would you have liked to have been offered this STAK programme as part of your child’s usual care? (advice and guidance around physical activity).

Yes No

Probe further: (if appropriate) At what stage after diagnosis would this have been useful?

1. Do you think that this intervention that promotes and supports children’s physical activity should be routinely offered to children when they are diagnosed with Type 1 Diabetes?

Yes No

Why?

1. Is there anything else you would like to say about this research or the STAK programme?
2. Do you have any questions?

**INTERVENTION GROUP ONLY**

1. How much did you and your child use the STAK programme?
   1. How much of the information about physical activity did you already know?
   2. Was there anything that prevented you engaging with the STAK programme or engaging in it as much as you would have liked?
2. What is your opinion of the STAK programme and the things included in it?
   1. STAK diary
      1. Was it pitched right?
      2. Did your child use it?
      3. What about it did **you** like/not like?
      4. What about it did **your child** like/not like?
   2. Parents booklet
      1. What messages do you think we were trying to give to children and their parents?
      2. What about it did **you** like/not like?
      3. What about it did **your child** like/not like?
   3. Dance DVDs
      1. Did you child look at the DVD?
      2. What about it did **you** like/not like?
      3. What about it did **your child** like/not like?
   4. Pedometer
      1. Did your child wear it?
      2. What about it did **you** like/not like?
      3. What about it did **your child** like/not like?
   5. Group sessions
      1. Did your child attend?
      2. Probe why they did/did not attend (e.g. location, time)
      3. What did you like about the group sessions (or idea of a group if they could not attend)?
      4. What did you not like about the group session?
      5. What about it did **your child** like/not like?
      6. What, if any, changes could we make to the group sessions to make them more appealing to your child?
   6. Your child will set some physical activity goals with Helen. What do you think about this?
3. What did you think was particularly good about the STAK programme?
   1. Content
   2. Format
4. What difficulties, if any, did you have with the STAK programme?
5. What would you change about the STAK programme to improve it?
   1. What additional information, if any, should be included?
   2. Content
   3. Format (e.g. paper diary)
6. Can you suggest other ways that could be used to provide information about physical activity for children with Type 1 diabetes and their parents?
7. What impact or effect, if any, did the STAK programme have on your child or family life?
8. Would you recommend this intervention to other families who have a child with Type 1 Diabetes?

Yes No

Probe further: why? / how come?

1. Is there anything else you would like to say about this research or the STAK programme?
2. Do you have any questions?
