# Supplementary material for: A physical activity intervention for children with type 1 diabetes- steps to active kids with diabetes (STAK-D): a feasibility study
Source: BMC Pediatr. 2018 Feb 7;18:37. doi: 10.1186/s12887-018-1036-8 (PMC5804086; doi:10.1186/s12887-018-1036-8)
Supplement: Supplementary file 4 — Post intervention qualitative interview guide VOLUNTEERS. (DOC 33 kb) [file 12887_2018_1036_MOESM4_ESM.doc]

**Steps To Active Kids (STAK) Programme: Feasibility Study**

**Qualitative Script: Acceptability, Desirability and Feasibility**

**Volunteers**

Participant ID……………………………………….

Date of Interview………………………………….

**** RECEIVE CONSENT BEFORE STARTING THE INTERVIEW ****

- - Have you read the information sheet?
  - Have you had the chance to ask any questions?
  - Are you happy to go ahead with the interview?

1. What are you studying – what year?
2. Overall, what do you think about this research?
   1. What attracted you to being involved?
3. What did you think about the training?
   1. Did you find it beneficial?
4. What do you think about the STAK group sessions?
5. How do you think the group sessions could have been done differently?
6. Attendance – suggestions for how to boost attendance rate?
   1. Could we have done anything differently?
7. What would you change about the group session to make it better?
   1. Activities
   2. Logistics – location/timing
8. Have you learnt anything from being involved in this project?
9. Would you recommend this intervention?

Yes No

Why?

What stage of diagnosis / age of child?

Anything else you would like to say about this research or the STAK programme?
