# Supplementary material for: A physical activity intervention for children with type 1 diabetes- steps to active kids with diabetes (STAK-D): a feasibility study
Source: BMC Pediatr. 2018 Feb 7;18:37. doi: 10.1186/s12887-018-1036-8 (PMC5804086; doi:10.1186/s12887-018-1036-8)
Supplement: Supplementary file 5 — Quotes to support the themes in the embedded qualitative study. (DOCX 17 kb) [file 12887_2018_1036_MOESM5_ESM.docx]

Additional File 5 Quotes to support the themes in the embedded qualitative study

| Theme and description | Quote | Respondent |
| --- | --- | --- |
| **Recruitment**: Preferred method of recruitment | I probably would’ve just left [a letter] to one side and not bothered. Whereas actually meeting [the researcher] in clinic encouraged us. | P07, mother, intervention group |
| **Recruitment**: Motivation for recruitment | To know how active I am. | P07, male, intervention group |
| **Recruitment**: Motivation for recruitment | To know I’m healthy. | P12, male, control group |
| **Recruitment**: Motivation for recruitment | You can track how much exercise he is doing, so I think that’s interesting to see how that affects him and blood sugar levels. | P08, mother, control group |
| **Recruitment**: Motivation for recruitment | I’m always up for anything which encourages [son] to be active and think about being active and just liked the idea that you were trying to see how kids responded to activity. | P04, mother, control group |
| **Recruitment**: Motivation for recruitment | Anything that can help another child with diabetes live a normal life. | P01, father, intervention group |
| **Recruitment**: Motivation for recruitment | [children with T1DM/their parents] take part in a lot of research, it’s like some felt like they’d done their bit, been bombarded over the years with research and had enough of filling forms in. | VOL08, clinical support worker |
| **Recruitment:**  Suggestions for improvement | [the research] had a good set-up, but the recruitment wasn’t great, so it’s whether as a clinic we could have done more… As a team we didn’t do enough. If we mention [research] in clinic, [patients] are usually quite excited and more than happy to do it. | HCP03, dietician |
| **Adherence:** barriers to attendance at group sessions | The 9 to 11 age group, they’re very dependent on parents actually taking them, they can’t make their own way there. Which could be a plus, it could be a negative. It just really shows it’s a parental engagement issue. | HCP01, consultant paediatrician |
| **Adherence**: barriers to attendance at group sessions | You’ve got to be incredibly dedicated and it just depends what kind of week you’ve had. And as much as you put 110% in to your kids [diabetes] management sometimes it’s just like, I don’t know if I can do this this weekend! | P02, mother, intervention group |
| **Adherence:** facilitators to continued participation | Because [STAK-D] it’s fun… I’m going to carry on. | P09, female, intervention group |
| **Adherence**: facilitators to continued participation | [bringing a friend or sibling to group sessions] created a bit of a division when some children didn’t have anyone [to bring]… That kid, her friend couldn’t come, she was really quite sad about it. | VOL02, group session volunteer |
| **Data completion:** Accelerometer | It was fun, but uncomfortable. | P06, female, intervention group |
| **Data completion**: Accelerometer | It was itchy. | P10, male, control group |
| **Data completion**: Accelerometer | It was too big, kept slipping. | P04, male, control group |
| **Data completion:** Accelerometer | It was alright but I couldn’t put water on it, so had to be careful. | P09, female, intervention group |
| **Intervention:** Information | It was stuff I already knew, but I think for somebody that probably has not dealt with diabetes before it would be really helpful. | P01, father, intervention group |
| **Intervention:** Pedometer | Trying to set more targets to get more steps on it so she was trying to find all different ways of doing it. | P01, mother, intervention group |
| **Intervention:** Pedometer | I was just looking at [son’s activity log] of the weeks he wrote [step count] down. And at school on average it was 7-8 thousand steps and I noticed on last Sunday he’d only done two and half thousand and that’s a huge difference, no wonder we have to up his basal [insulin dose] by 20%. It supports your management of your child when you can see the evidence in front of you. | P02, mother, intervention group |
| **Intervention**: Group activity session | I tried things that I haven’t tried before like the rowing and step-ups and the big ball against the wall to squat. | P09, female, intervention group |
| **Intervention**: Group activity session | I had fun doing active stuff…and I could spend time with my friend. | P07, male, intervention group |
| **Intervention**: perceived benefits | What was really useful from your feedback was finding out what was really happening with young people despite the multiple pieces of advice we give them and what they say they do, in reality what was really going on and it in no way matched what they were advised to do in an shape or form. So that was really interesting and useful… perhaps we’ve unmasked what really goes on in the real life rather than when they come into clinic. | HCP01, consultant paediatrician |
| **Intervention**: perceived benefits | Some families are completely against meeting other people with diabetes… and vice versa there are people that would really like social interaction. | HCP03, dietician |
| **Intervention**: perceived benefits | It was really good that there was a parents’ information leaflet that they could fill in and feel involved in. So it was their responsibility to make sure that their child was active, rather than assuming their child would go away and do activity without being motivated or encouraged to do it. | HCP03, dietician |
| **Intervention**: perceived benefits | As a family, the other daughter as well does the exercise especially the STAK DVDs. | P09, father, intervention group |
| **Intervention**: perceived benefits | One thing we did do was get back on the Wii Fit and the Wii Sport which we hadn’t done in a while. | P02, mother, intervention group |
